# FXTAS is difficult to differentiate from neuronal intranuclear inclusion disease through skin biopsy: a case report

**DOI:** 10.1186/s12883-021-02425-z

**Published:** 2021-10-12

**Authors:** Megumi Toko, Tomohiko Ohshita, Takashi Kurashige, Hiroyuki Morino, Kodai Kume, Hiroshi Yamashita, Gen Sobue, Yasushi Iwasaki, Jun Sone, Hideshi Kawakami, Hirofumi Maruyama

**Affiliations:** 1 Department of Neurology, Hiroshima City Asa Citizens Hospital, 2-1-1, Kabeminami, Asakita-ku, Hiroshima, 731-0293 Japan; 2 Department of Clinical Neuroscience and Therapeutics, Hiroshima University Graduate School of Biomedical and Health Sciences, 1-2-3 Kasumi, Minami-ku, Hiroshima, 734-8553 Japan; 3 Department of Neurology, National Hospital Organization Kure Medical Center and Chugoku Caner Center, 3-1 Aoyama-cho, Kure, Hiroshima, 737-0023 Japan; 4 Department of Epidemiology, Research Institute for Radiation Biology and Medicine, Hiroshima University, 1-2-3 Kasumi, Minami-ku, Hiroshima, 734-8553 Japan; 5 Department of Supportive and Promotive Medicine of the Municipal Hospital, Faculty of Medicine, Kagawa University, 1750-1 Ikenobe, Miki-cho, Kita-gun, Kagawa 761-0793 Japan; 6 Aichi Medical University, Nagakute, Aichi Japan; 7 Department of Neuropathology, Institute for Medical Science of Aging, Aichi Medical University, Nagakute, Aichi 480-1195 Japan; 8 Department of Neurology, National Hospital Organization Suzuka National Hospital, 3-2-1, Kasado, Suzuka, Mie 513-8501 Japan

**Keywords:** FXTAS, NIID, Skin biopsy, Genetic analysis

## Abstract

**Background:**

Both fragile X-associated tremor/ataxia syndrome (FXTAS) and late-onset neuronal intranuclear inclusion disease (NIID) show CGG/GGC trinucleotide repeat expansions. Differentiating these diseases are difficult because of the similarity in their clinical and radiological features. It is unclear that skin biopsy can distinguish NIID from FXTAS. We performed a skin biopsy in an FXTAS case with cognitive dysfunction and peripheral neuropathy without tremor, which was initially suspected to be NIID.

**Case presentation:**

The patient underwent neurological assessment and examinations, including laboratory tests, electrophysiologic test, imaging, skin biopsy, and genetic test. A brain MRI showed hyperintensity lesions along the corticomedullary junction on diffusion-weighted imaging (DWI) in addition to middle cerebellar peduncle sign (MCP sign). We suspected NIID from the clinical picture and the radiological findings, and performed a skin biopsy. The skin biopsy specimen showed ubiquitin- and p62-positive intranuclear inclusions, suggesting NIID. However, a genetic analysis for NIID using repeat-primed polymerase chain reaction (RP-PCR) revealed no expansion detected in the *Notch 2 N-terminal like C* (*NOTCH2NLC*) gene*.* We then performed genetic analysis for FXTAS using RP-PCR, which revealed a repeat CGG/GGC expansion in the *FMRP translational regulator 1* (*FMR1*) gene. The number of repeats was 83. We finally diagnosed the patient with FXTAS rather than NIID.

**Conclusions:**

For the differential diagnosis of FXTAS and NIID, a skin biopsy alone is insufficient; instead, genetic analysis, is essential. Further investigations in additional cases based on genetic analysis are needed to elucidate the clinical and pathological differences between FXTAS and NIID.

## Background

Fragile X-associated tremor/ataxia syndrome (FXTAS) is a late-onset inherited degenerative disorder due to a CGG repeat expansion in the premutation range (55–200) in the 5′ untranslated region of the *FMRP translational regulator 1* (*FMR1*) gene [1]. FXTAS is an important differential diagnosis for late-onset neuronal intranuclear inclusion disease (NIID), because both are similar in their clinical and radiological features. Recently, a genetic abnormality, a GGC repeat expansion in the 5′ untranslated region of *Notch 2 N-terminal like C* (*NOTCH2NLC*), was revealed in NIID [2]. These disorders were hypothesized to share a similar molecular basis caused by noncoding CGG/GGC trinucleotide repeat expansions. Pathological studies in autopsy cases revealed that both diseases showed the ubiquitin-positive intranuclear inclusion exhibited throughout the brain and periphery. For the diagnosis of NIID, skin biopsy was reported as a useful method [3]. However, it is unclear that skin biopsy can distinguish NIID from FXTAS because no report has described the pathological findings from a skin biopsy in the patients with FXTAS.

We performed skin biopsy in a patient with FXTAS with cognitive dysfunction and peripheral neuropathy without tremor who was suspected to be NIID at first and was finally diagnosed as FXTAS owing to genetic analysis.

## Case presentation

**Fig. 1:**
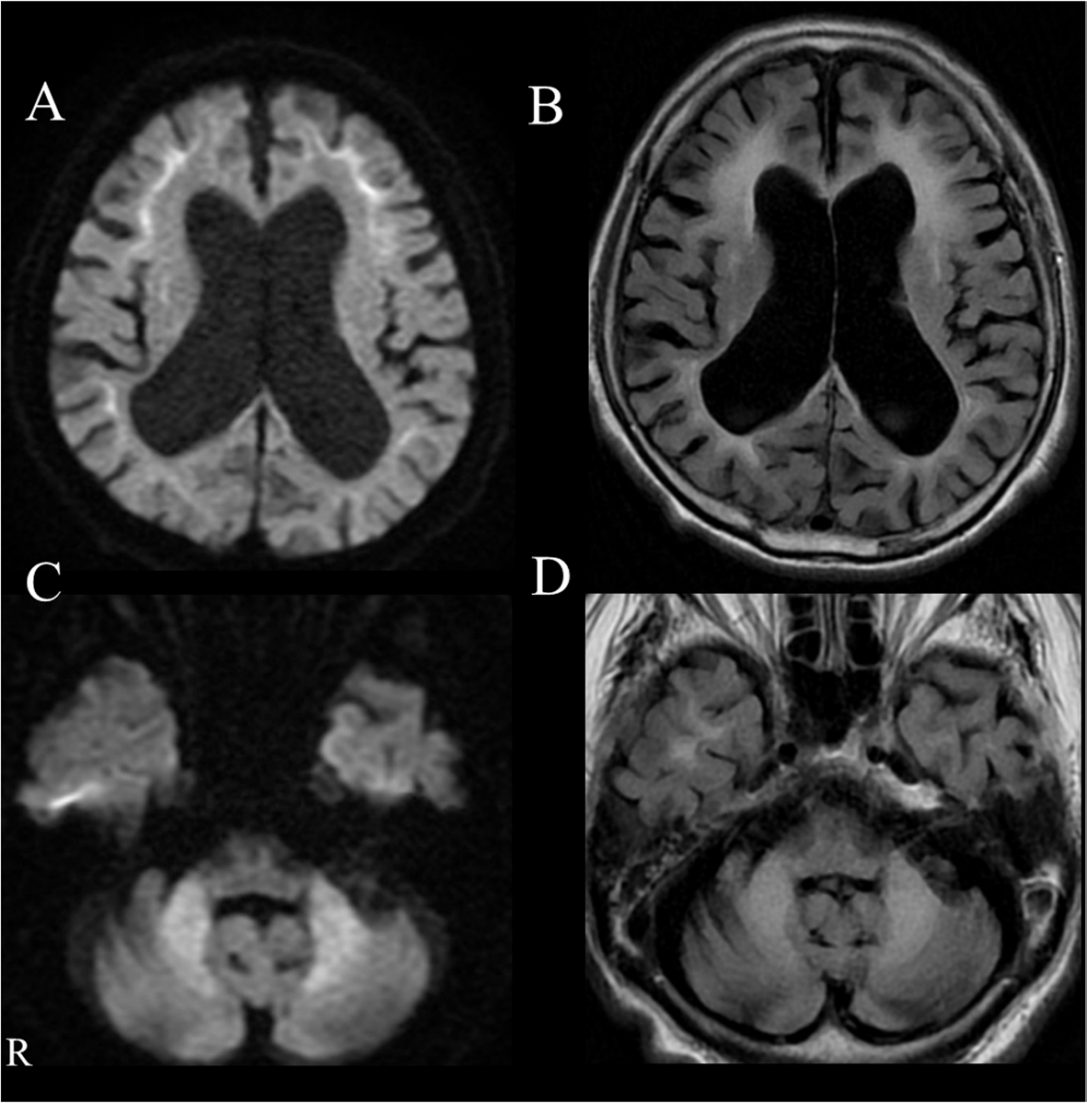
**A**–**D** Head MRI findings; an axial DWI showing high signal intensity along the corticomedullary junction (**A**). An axial FLAIR image showing diffuse atrophy of the cerebrum and increased signal intensity in the cerebral white matter (**B**). Bilateral abnormal high signal intensity in the middle cerebellar peduncles (MCP sign) is shown on axial DWI (**C**) and FLAIR (**D**)

**Fig. 2:**
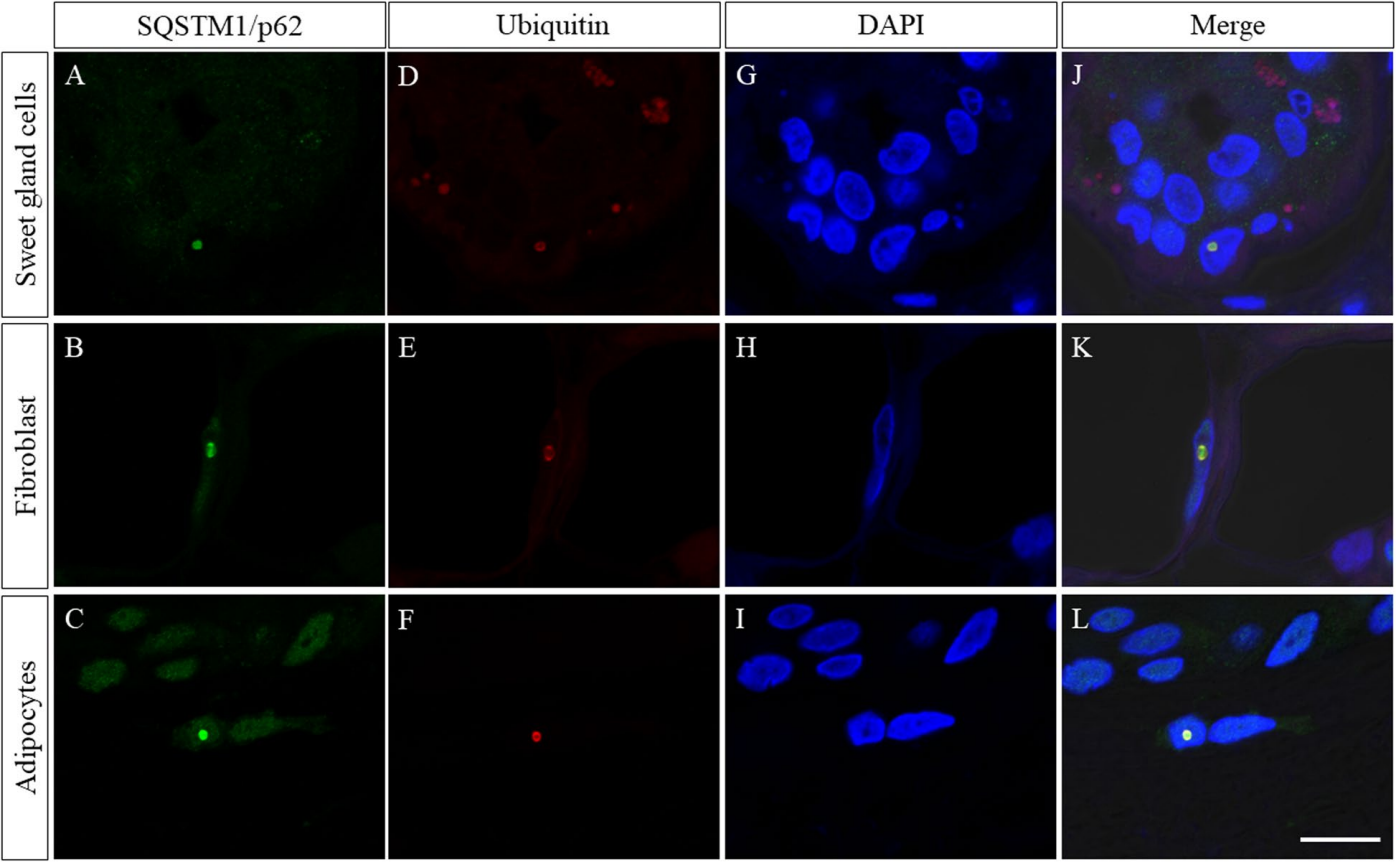
Immunofluorescent findings of the skin biopsy specimen; skin biopsy specimen showed p62-positive aggregates (**A**–**C**) and ubiquitin-positive aggregates (**D**–**F**) in sweat gland cells (upper), fibroblasts (middle), and adipocytes (lower). Immunofluorescent analysis with 4′,6-diamidino-2-phenyindole di-lactate (DAPI) (**G**–**I**) revealed that these aggregates were co-localized in the nuclei stained with DAPI (**J**–**L**). Scale bar = 10 μm

A patient in his 70s, with a history of type 2 diabetes mellitus and bronchial asthma, presented with progressive gait disturbance and cognitive impairment in 2017. He did not have a family history of neurological disease. On admission, we did not detect any problems from the general physical examination including consciousness and body temperature. He showed general cognitive impairment, yielding a Mini-Mental State Examination score of 13/30. His ocular movements showed saccadic pursuits without nystagmus. Additionally, muscle weakness was observed in the bilateral lower extremities, predominantly on the right side. In the bilateral lower extremities, vibration and proprioceptive sensations were decreased and deep tendon reflexes were absent. He also showed limb ataxia but no tremor. Autonomic dysfunction was not observed. A blood test including hemoglobin A1c, thyroid hormone, and autoantibodies (anti-SS-A antibody, anti-SS-B antibody, proteinase-3-anti-neutrophil cytoplasmic antibody, myeloperoxidase-anti-neutrophil cytoplasmic antibody, anti-Aquaporin 4 antibody, and anti-double stranded DNA IgG antibody) showed no abnormalities that could explain his symptoms. An examination of cerebrospinal fluid showed a slight increase in protein (59 mg/dL). A nerve conduction study showed reduced compound muscle action potential amplitude and reduced sensory potentials in the upper and lower limb nerves. An EEG showed general theta waves with no epileptic waves. Therefore, we performed brain MRI and observed hyperintensity lesions along the corticomedullary junction on diffusion-weighted imaging (DWI) in addition to middle cerebellar peduncle sign (MCP sign) (Fig. 1). ^123^I-iodoamphetamine SPECT showed reduction of blood flow in the bilateral cerebellum hemisphere. Considering the clinical picture which showed cognitive impairment and no tremor, as well as the corticomedullary hyperintensity lesions on DWI, we initially suspected NIID, although FXTAS had to be differentiated. In the diagnostic flow chart of adult onset NIID reported by Sone et al., skin biopsy is recommended as the next step after the electrophysiological, radiological, and biochemical examinations [4]. Thus, we performed a skin biopsy from the abdominal wall. The skin biopsy specimen showed sparse p62-positive aggregates and ubiquitin-positive aggregates co-localized in the nuclei stained with 4′,6-diamidino-2-phenyindole di-lactate in adipocytes, sweat gland cells, and dermal fibroblasts (Fig. 2), further suggesting NIID.

**Fig. 3:**
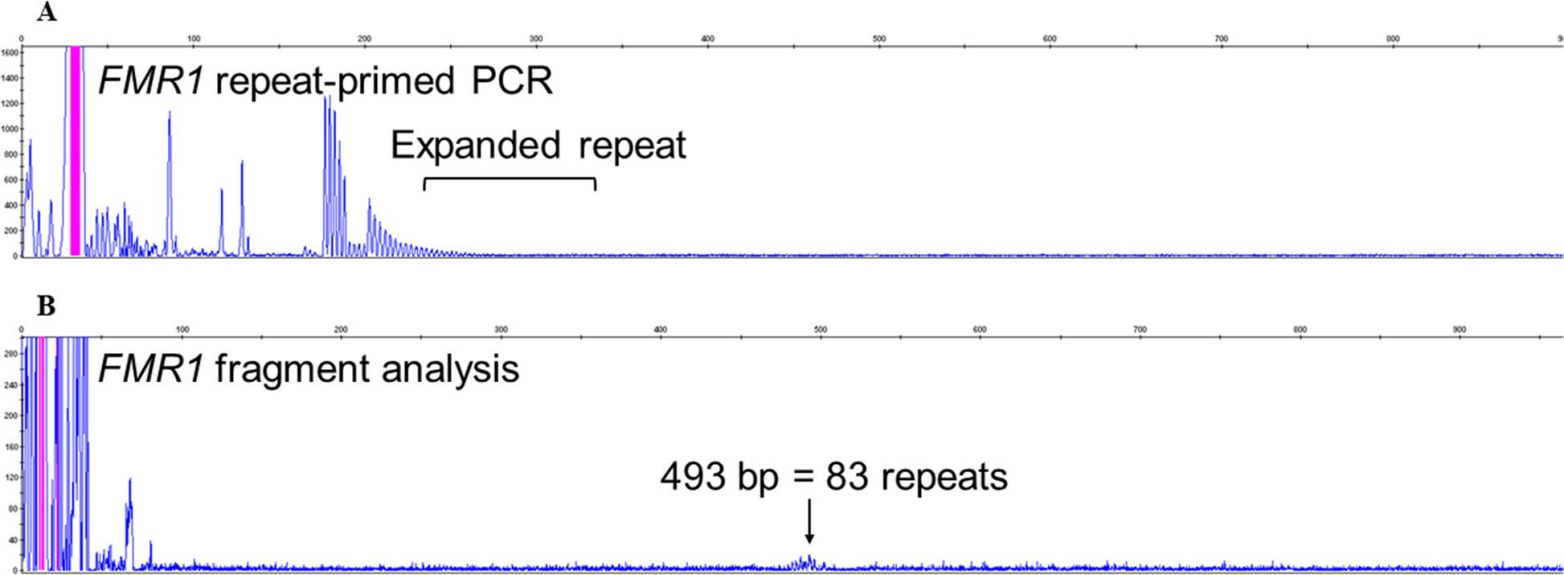
**A**–**B** Genetic analysis; repeat-primed polymerase chain reaction (RP-PCR) showed repeat expansion of *FMR1* gene (**A**). The number of repeats was calculated by fragment analysis, and the patient had 83 repeats (**B**)

With the report of a genetic cause of NIID in 2019, we performed a genetic analysis for NIID using repeat-primed polymerase chain reaction (RP-PCR) for confirmation of diagnosis, using genomic DNA extracted from peripheral blood leukocytes as previously reported [2]. The RP-PCR revealed no expansion in the *NOTCH2NLC* gene*.* Taking this result, we then performed genetic analysis for FXTAS using RP-PCR with genomic DNA extracted from peripheral blood leukocytes with reference to a past report [5]. As a result, a repeat CGG/GGC expansion in the *FMR1* gene was revealed (Fig. 3A). The number of repeats was calculated by fragment analysis, and the patient had 83 repeats (Fig. 3B). Therefore, we diagnosed the patient with FXTAS rather than NIID.

## Discussion and conclusions

Both FXTAS and NIID show cerebellar ataxia, dementia, and neuropathy. The tremor can be unremarkable in FXTAS as was the case with our patient.

Importantly, an MRI of FXTAS usually demonstrates cerebral and cerebellar atrophy, increased T2 signal intensity in the cerebral and cerebellar white matters, and an MCP sign. An MCP sign is a well-known characteristic of FXTAS, which we observed in our patient; however, it has also been reported in patients with NIID [6]. Furthermore, while high-intensity signals on DWI in the corticomedullary junction are known as one of the characteristic features of NIID [3], they have also been previously observed in FXTAS patients [6]. Taken together, this suggests that it is not possible to accurately diagnose the patient based on these findings alone.

The pathological features of FXTAS in autopsy cases include ubiquitin-positive intranuclear inclusions exhibited in neurons and astrocytes throughout the brain and periphery [7, 8]. The size, shape, component, and distribution of inclusions in FXTAS resemble those of NIID [4]. However, previous studies have not reported any dermal pathological findings in either autopsy cases or cases with skin biopsy. In this FXTAS patient, we detected ubiquitin- and p62-positive intranuclear inclusions similar to those found in NIID patients, although the number of inclusions was small. We did not analyze Heat Shock protein 70 (HSP 70), which has been demonstrated to increase in FXTAS fibroblasts [9]. To distinguish FXTAS from NIID using these data from skin biopsy was difficult. More detailed analysis including HSP 70 and electron microscopy and further study with a greater number of patients may confirm skin biopsy as a key tool for differential diagnosis.

Finally, we were able to diagnose FXTAS by genetic analysis. This finding suggests that a skin biopsy alone is insufficient and that, instead, genetic analysis is essential for the differential diagnosis of FXTAS and NIID. Further investigations in additional cases based on genetic analysis are needed to elucidate the clinical and pathological differences between FXTAS and NIID.

## Data Availability

All data generated or analyzed during this study are included in this published article.

## Abbreviations

FXTAS: Fragile X-associated tremor/ataxia syndrome
NIID: Late-onset neuronal intranuclear inclusion disease
DWI: Diffusion-weighted imaging
MCP sign: Middle cerebellar peduncle sign
*NOTCH2NLC*: *Notch 2 N-terminal like C*
*FMR1*: *FMRP translational regulator 1*
RP-PCR: Repeat-primed polymerase chain reaction; HSP 70: Heat Shock protein 70
