# Correction to: FXTAS is difficult to differentiate from neuronal intranuclear inclusion disease through skin biopsy: a case report

**DOI:** 10.1186/s12883-021-02452-w

**Published:** 2021-10-27

**Authors:** Megumi Toko, Tomohiko Ohshita, Takashi Kurashige, Hiroyuki Morino, Kodai Kume, Hiroshi Yamashita, Gen Sobue, Yasushi Iwasaki, Jun Sone, Hideshi Kawakami, Hirofumi Maruyama

**Affiliations:** 1 Department of Neurology, Hiroshima City Asa Citizens Hospital, 2–1-1, Kabeminami, Asakita-ku, Hiroshima, 731–0293 Japan; 2 Department of Clinical Neuroscience and Therapeutics, Hiroshima University Graduate School of Biomedical and Health Sciences, 1–2-3 Kasumi, Minami-ku, Hiroshima, 734–8553 Japan; 3 Department of Neurology, National Hospital Organization Kure Medical Center and Chugoku Caner Center, 3–1 Aoyama-cho, Kure, Hiroshima, 737–0023 Japan; 4 Department of Epidemiology, Research Institute for Radiation Biology and Medicine, Hiroshima University, 1–2-3 Kasumi, Minami-ku, Hiroshima, 734–8553 Japan; 5 Department of Supportive and Promotive Medicine of the Municipal Hospital, Faculty of Medicine, Kagawa University, 1750–1 Ikenobe Kagawa, Miki-cho, Kita-gun 761–0793 Japan; 6 Aichi Medical University, Nagakute, Aichi Japan; 7 Department of Neuropathology, Institute for Medical Science of Aging, Aichi Medical University, Nagakute, Aichi 480–1195 Japan; 8 Department of Neurology, National Hospital Organization Suzuka National Hospital, 3–2-1, Kasado, Suzuka, Mie 513–8501 Japan

**Correction to: BMC Neurol 21, 396 (2021)**

**https://doi.org/10.1186/s12883-021-02425-z**

Following publication of the original article [1], the authors reported that the captured corresponding author is not correct. The correct Corresponding author should be “Tomohiko Ohshita” and not “Megumi Toko”.

The original article [1] has been updated.
